# The role of soluble suppression of tumorigenicity 2 in pulmonary hypertension: a systematic review and meta-analysis

**DOI:** 10.1016/j.ahjo.2026.100827

**Published:** 2026-07-02

**Authors:** Liuzhao Cao, Weiyun Teng, Linli Sang, Xingxiang Xu

**Affiliations:** Northern Jiangsu People's Hospital affiliated to Yangzhou University, People's Republic of China

**Keywords:** Soluble suppression of tumorigenicity 2, Pulmonary hypertension, Pulmonary arterial pressure, Biomarkers

## Abstract

**Background:**

Pulmonary hypertension (pH) is characterized by an increase in pulmonary vascular resistance. Although soluble suppression of tumorigenicity-2 (sST2) has been identified as a biomarker for heart failure, the implication of sST2 in PH has not been well studied.

**Objective:**

This study aimed to investigate the value of sST2 in predicting prognosis of PH and the correlation between sST2 and pulmonary arterial pressure (PAP).

**Methods:**

We conducted a systematic review of databases including PubMed and Embase. Levels of sST2 in the PH group and the control group were compared. Data on the accuracy of sST2 for predicting clinical worsening and Hazard ratios (HR) for the association of sST2 with poor outcomes were pooled. Additionally, correlation coefficients between sST2 levels and mean pulmonary arterial pressure (mPAP) and several other clinical parameters were pooled. Meta-regression and subgroup analysis was conducted to identify the source of heterogeneity.

**Results:**

15 studies were included in our study. Levels of sST2 were significantly higher in PH group (641 subjects) than control group (575 subjects) (SMD 0.71, 95%CI 0.43–0.98, *P* < 0.001). Among 352 patients, the sST2 had a pooled sensitivity of 0.92 (95% CI 0.69–0.99) and specificity of 0.62 (95%CI 0.42–0.79) in predicting clinical worsening. The Summary Receiver Operating Characteristic (SROC) curve of sST2 was plotted and area under curve (AUC) was 0.82 (95%CI 0.79–0.85). Elevated sST2 levels were significantly associated with poor outcomes (HR: 1.92, 95% CI: 1.09–3.36). The HR was derived from the log2 HR (log_2_ HR: 0.94, 95%CI 0.12–1.75). SST2 levels were significantly relative to PAP and the pooled correlation coefficient was 0.25 (95%CI 0.16–0.35). Subgroups analysis demonstrated that classification of PH was probably the source of heterogeneity.

**Conclusion:**

SST2 levels were elevated among patients with PH and correlated to PAP and right cardiac dysfunction. sST2 played a reliable role in predicting prognosis of PH despite the limitations and heterogeneity among the studies.

## 1 Introduction

Pulmonary hypertension (pH) is a pathophysiological condition characterized by an increase in pulmonary vascular resistance and elevated pulmonary arterial pressure (PAP) [1]. These changes result in an increased load on the right heart, which can ultimately lead to right heart failure. PH can be idiopathic or secondary to other underlying conditions such as lung diseases, connective tissue disorders, congenital heart defects, or left heart disease. Five main groups of PH are recognized including idiopathic pulmonary arterial hypertension (PAH), pH associated with left heart disease, pH associated with lung disease or hypoxemia, pH associated with pulmonary artery obstructions, and PH with unclear and/or multifactorial mechanisms [1]. The symptoms of pulmonary hypertension including shortness of breath during activity, fatigue and an increased heart rate are commonly non-specific [2]. If not diagnosed promptly and treated effectively, pH can lead to a poor prognosis. However, there is a lack of a reliable biomarker to evaluate the prognosis during follow-up [3].

Soluble Suppression of Tumorigenicity 2 (sST2), functioning as the receptor for interleukin-33 (IL-33), is an increasingly recognized biomarker due to its role in inflammation and cardiovascular [4], [5]. Mechanical stress placed on the heart by PH may increase the expression of sST2. The levels of sST2 appear to relate to pulmonary vascular resistance, cardiac index, and clinical deterioration [6]. Although several studies have reported an elevated level of sST2 in patients with PH [7], the correlation between sST2 level and pulmonary arterial pressure, as well as its value in predicting prognosis of PH, remained unclear. Therefore, we conducted a systemic review and meta-analysis on the correlation between sST2 level and pulmonary arterial pressure. This study explored the prognostic potential of sST2 in predicting the outcomes of PH as well.

## 2 Methods

### 2.1 Search strategy

**Fig. 1:**
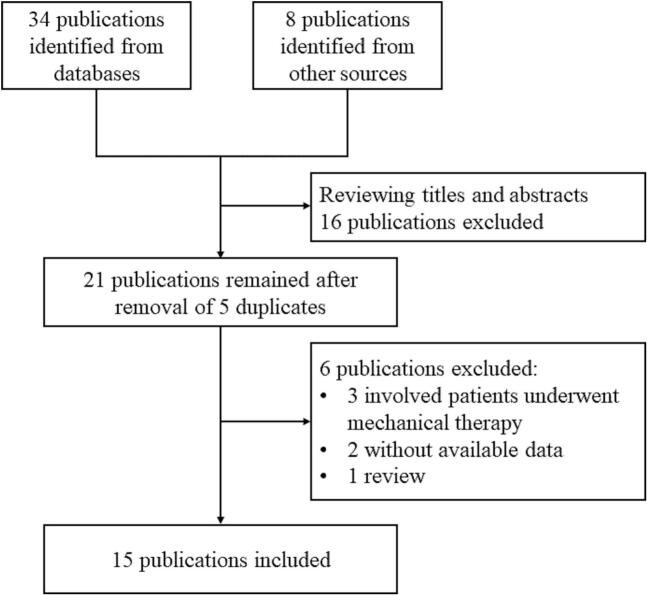
Selection of trials in this meta-analysis.

Our study has registered on PROSPERO database (Record ID: CRD42024529503). Two independent investigators searched PubMed and Embase databases (from inception to February 2024), with no language or region restrictions. Potential eligible trials were also screened from other sources. The following keywords were used for searching: ‘Soluble Suppression of Tumorigenicity 2’, ‘sST2’, ‘pulmonary hypertension’, and ‘PH’. The search results were merged, and duplicate records were removed. One reviewer scanned the titles and abstracts to identify potential eligible articles and ruled out the irrelevant articles. Two reviewers retrieved the potentially relevant studies then went through the full texts and independently extracted the data. If there were any different opinions, the third reviewer made the final decision. The flow chart was shown in Fig. 1. This study is a meta-analysis and does not involve the collection of new original data or experiments; therefore, it does not require approval from an ethics committee or informed consent from participants.

### 2.2 Inclusion and exclusion criteria

Inclusion and exclusion criteria for the study were as follows: The inclusion encompassed studies that reported sST2 values and investigated the correlation between sST2 levels and PAP in adult subjects with PH, as well as the association between sST2 levels and clinical outcomes. Outcomes defined by each study including death, heart failure, readmission and lung transplantation. This study excluded post-intervention data from non-pharmacological treatments, and included only pre-treatment biomarker data. Reviews, case reports, articles without sufficient data, and researches involving children were not included as well.

### 2.3 Definition

Diagnosis of PH was made in the presence of a resting mean pulmonary artery pressure (mPAP) of >25 mmHg with Pulmonary vascular resistance (PVR) >2 WU and pulmonary artery wedge pressure (PAWP) ≤15 mmHg measured upon Right Heart Catheterization (RHC) or pulmonary artery systolic pressure (PASP) ≥35 mmHg assessed by echocardiography. The control group was defined by the studies involved as the absence of a PH diagnosis or healthy volunteers. The clinical worsening was defined as death and readmission for worsening PH including heart failure, lung or heart/lung transplant or unsatisfactory clinical response. The incidence of clinical worsening and mortality in the studies included were listed in Table 2.

### 2.4 Data extraction

Data was independently extracted by two reviewers, including details such as the first author's name, publication year, country, number of subjects, patient category, study design, methods for measuring PAP and the detection method of sST2. The characteristics of the selected studies were summarized in Table 1. Characteristic of subjects, demographics, results of mPAP or PASP and sST2 in each group, cutoff value of sST2 and methods to determine cutoff value were pooled in the Table 2 as well. The accuracy of the extracted data was verified independently by two reviewers, with any disagreements resolved through discussion with a third reviewer. Mean differences (MD) in sST2 levels between groups (subjects with or without PH, and subjects with or without clinical worsening) were calculated and pooled for our meta-analysis. For the correlation between sST2 levels and PAP, Pearson correlation coefficients (r) with 95% confidence intervals (CI) were extracted and analyzed. Hazard ratios (HR) with 95% CIs were extracted and pooled to assess the relationship between levels of sST2 and clinical outcomes. In studies that evaluated predictive value using ROC curves, cutoff values were also extracted, and a pool of sensitivity and specificity was analyzed.

**Table 1:** Main characteristics of the studies included in the systematic review.

Author (Year)	Year	Nation	NOS scores	sST2 detection methods	PAP detection methods	Study design
Pratama RS [8]	2020	Indonesia	6	Cartridge-based immunofluorescence	RHC	prospective observational cohort study
Yang HS [9]	2016	Italy	5	ELISA	TTE	retrospective cohort study
Sun Y [10]	2021	China	8	Sandwich monoclonal immunoassay	RHC	retrospective cohort study
Boxhammer E [11]	2022	Germany	9	ELISA	RHC	prospective observational cohort study
Lv Z [12]	2023	China	7	ELISA	TTE	prospective observational cohort study
Yu YZ [13]	2022	China	6	Sandwich monoclonal immunoassay	TTE	retrospective cohort study
Mima M [7]	2019	Germany	5	ELISA	RHC	retrospective cohort study
Ye HS [14]	2022	China	8	-	TTE	retrospective cohort study
Simpson CE [15]	2020	America	8	ELISA	RHC	multi-centers prospective observational cohort study
Geenen LW [16]	2019	Netherlands	9	ELISA	RHC	prospective observational cohort study
Kerkütlüoğlu M [17]	2023	Turkey	7	Sandwich monoclonal immunoassay	TTE	prospective observational cohort study
Carlomagno G [18]	2013	Italy	5	ELISA	RHC	prospective observational cohort study
Plácido R [19]	2017	France	9	Chemiluminescence assays	TTE	prospective observational cohort study
Zheng YG [21]	2014	China	9	ELISA	RHC	prospective observational cohort study
Agoston-Coldea L [22]	2014	Romania	7	ELISA	TTE	prospective observational cohort study

PH: Pulmonary hypertension; PAP: Pulmonary arterial pressure; sST2: Soluble Suppression of Tumorigenicity 2; NOS: Newcastle-Ottawa Scale; TTE: Transthoracic echocardiography; ELISA: Enzyme-linked immunosorbent assay; RHC: Right heart catheterization.

**Table 2:** Characteristics of the subjects enrolled in each study.

Author (Year)	Subjects with PH	Subjects of control goup	N of PH	N of control	Age of PH	Age of control	Overall male, N(%)	PAP of PH, mmHg	sST2, ng/mL (PH vs control)	Cut-off of sST2, ng/mL	Optimal Cut-off Value Determination Method	Definition of poor prognosis	Percentage of poor prognosis	mortality
Pratama RS (2020) [8]	Uncorrected ASD	ASD with no PH	81	18	36.7±13.9	30.1±10.6	12(14.8)	44.0±20.7	22.30±15.07 vs 19.24±11.99	-				
Yang HS (2016) [9]	Sepsis	-	127	-	71 (58-80)		70 (55.0)	29.4(24.4, 37.0)	110.1 (54.2, 240.1)	-				
Sun Y (2021) [10]	COPD IPAH CTEPH	Healthy controls	184	14	51.4±18.5	50.2±17.9	91(46.0)	49.5(30.0, 63.8)	33.1 (26.6, 46.3)	30.4	Youden index.	Death	35.3%	35.3%
Boxhammer E (2022) [11]	Patients with severe AS undergoing TAVR	Patients diagnosed with severe AS and no PH.	109	43	81.05±7.6	80.91±6.87	-	38.35±10.27	8.24±8.19 vs 5.23±3.63	-				
Lv Z (2023) [12]	PH secondary to AECOPD	AECOPD without PH	57	69	73.00 (65.50, 83.00)	72.00 (62.50, 79.00)	104(82.6)	-	69.67±21.11 vs 59.50±32.10	59.1	Youden index.	Readmission within 90 days	63.2%	
Yu YZ (2022) [13]	IPF patients	Healthy controls	58	25	60.47±10.39	59.55±13.82	67(65.0)	-	-	-				
Mima M (2019) [7]	Group1,2,3,4,5	Patients admitted for elective coronary angiography with no coronary artery disease.	88	74	71±12.79	58±17.46	58(35.8)	41.96±10.76	10.14±14.75 vs 5.11±1.68	-				
Ye HS (2022) [14]	CTD-PH	CTD patients without PH	71	21	37.00 (29.00–55.00)	50.00(43.50-53.00)	3(3.3)	69.77±20.93	39.57±23.72 vs 17.96±12.13	39.99	Maximization of differences method	Death, hospitalization for worsening PH including lung or heart/lung transplant or unsatisfactory clinical response	36.6%	
Simpson CE (2020) [15]	Group 1	-	2017	-	55 (15)	-	406(20)	50±15	5.59 (3.77, 8.94)	5.59	Median value	Death	16.0%	16.0%
Geenen LW (2019) [16]	Group1,2,3,4,5	Healthy controls	104	142	59 (47–69)	-	38(36)	42 (35, 52)	27.9 (19.6, 44.9)	23.5	Median value	Death, lung transplantation or heart failure	58.7%	29.8%
Kerkütlüoğlu M (2023) [17]	CTEPH	Patients with PE who did not develop CTEPH	100	89	67 ± 10	65 ± 18	41(41)	46.55±7.85	209.00±51.23 vs 71.00±70.38	-				
Carlomagno G (2013) [18]	IPAH/SScl/other	Healthy contros	25	10	61±8	-		47±11	42.82±19.09 vs 14.84±1.90	43.3	Median value			
Plácido R (2017) [19]	Group1,3,4,5		43	-	59(43-67)	-	12(27.9)	64.6 (46.0, 84.7)	47.7 (27.1, 75.5)	68.6	Third tertile	Death or heart failure or readmission	41.9%	25.6%
Zheng YG (2014) [21]	Idiopathic PAH	Healthy volunteers.	40	24	32.0±10.9	30.4±7.5	11(17)	60.9±17.6	28.9±13.9 vs 20.7±7.5	34.1	Youden index.	Death, lung transplantation, hospitalization for PAH, the initiation of a new therapy, or worsening WHO functional class	30.0%	10.0%
Agoston-Coldea L (2014) [22]	PH secondary to COPD	Healthy controls	36	36	59+7	59+8	36(50)	57.1 ±9.8	1.60±1.91 vs 0.70±0.86	-				

PH: Pulmonary hypertension; PAP: Pulmonary arterial pressure; sST2: Soluble Suppression of Tumorigenicity 2; ASD: Atrial septal defect; AECOPD: Acute exacerbation of chronic obstructive pulmonary disease; IPAH: Idiopathic pulmonary arterial hypertension; CTEPH: Chronic thromboembolic pulmonary hypertension; PE: Pulmonary embolism; AS: Aortic valve stenosis; TAVR: Transcatheter aortic valve replacement; IPF: Idiopathic pulmonary fibrosis; CTD: Connective tissue disease; PAH: Pulmonary arterial hypertension; SScl: Systemic sclerosis.

### 2.5 Certainty of the evidence

The Grading of Recommendations, Assessment, Development, and Evaluation (GRADE) framework was also adopted to evaluate the certainty of the evidence in all outcomes, ranging from high quality to very low quality based on effect size. The evidence was upgraded or downgraded based on the GRADE criteria. Two authors (Cao and Teng) assessed the strength of evidence according to the Grading of Recommendations, Assessment, Development, and Evaluation (GRADE) criteria (Supplementary Table 1).

### 2.6 Risk of bias assessment

The quality of the studies included in our meta-analysis was evaluated using the Newcastle-Ottawa Quality Assessment Scale (NOS), which assessed studies across three key dimensions: study participant selection, comparability of results, and outcome integrity. The total scores of each study were listed in Table 1. The Quality in Prognostic Studies (QUIPS) tool was used to assess the confounding, attrition, and analysis domains in the prognostic studies, and the results were presented in a table in the supplementary material (Supplementary Table 2).

### 2.7 Statistical analysis

The PRISMA (Preferred Reporting for Systematic Reviews and Meta-Analysis) statement was followed when performing this meta-analysis. Statistical analysis was conducted using STATA software (version 15).

Standardized effect sizes (Hedge's g) with corresponding confidence intervals were calculated for the levels of sST2. Hierarchical Summary Receiver Operating Characteristic (HSROC) and bivariate model were constructed to evaluate the accuracy. Summary Receiver Operating Characteristic (SROC) curves were plotted to evaluate the true positive and false positive rates of each study. The exact area under the curve (AUC) of the SROC plot was indicative of the test's overall accuracy. A fixed-effect model was utilized when no significant heterogeneity was detected among the studies. A random-effects model was employed when heterogeneity was substantial, indicated by a *P*-value ≤0.1 and/or an I^2^ > 50%. A random-effects model was employed when substantial heterogeneity was observed, as indicated by a P-value ≤0.1, I^2^ > 50%, and/or τ^2^ > 0.04. The significance of the individual study effects was further assessed using z-values, with a threshold of *P* < 0.05 for statistical significance. To locate the origin of the heterogeneity, meta-regression was conducted and subgroup analysis based on different disease groups was performed. Funnel plots showing standard errors or precision against the logarithms of the odds ratio were constructed. Begg and Mazumdar rank correlation test and Egger's test were used to assess for possible publication bias. A trim-and-fill analysis was conducted to analysis the publication bias as well. Results were visually presented using forest plots. To assess the robustness of the pooled effect size, the leave-one-out method was performed to analyze the sensitivity. In this analysis, each study was sequentially excluded from the meta-analysis to determine its influence on the overall results. The effect size was recalculated, and the consistency of the results was evaluated to identify whether any single study had a disproportionate impact on the pooled effect size. A *P* < 0.05 was considered statistically significant.

## 3 Results

### 3.1 Studies inclusion

**Fig. 2:**
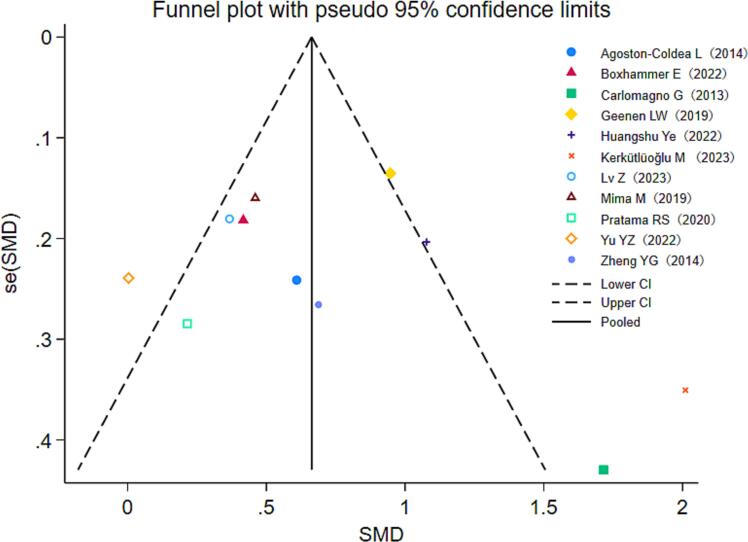
Funnel plot showed comparison ofsST2 levels between control and PH groups by standardized mean difference.

We identified 34 publications from databases and 8 from other sources which could be involved. 21 records were ruled out after reviewing abstracts and removing duplicates. After reviewing of full text and final resolution, 15 articles were involved in our meta-analysis (Fig. 1) [7], [8], [9], [10], [11], [12], [13], [14], [15], [16], [17], [18], [19], [21]. The main characteristics and NOS scores of each study were summarized and presented in Table 1. The characteristics of the subjects included in each study were detailed in Table 2. Funnel plot did not suggest obvious publication bias with visual inspection (Fig. 2).

### 3.2 Elevation of sST2 among PH

**Fig. 3:**
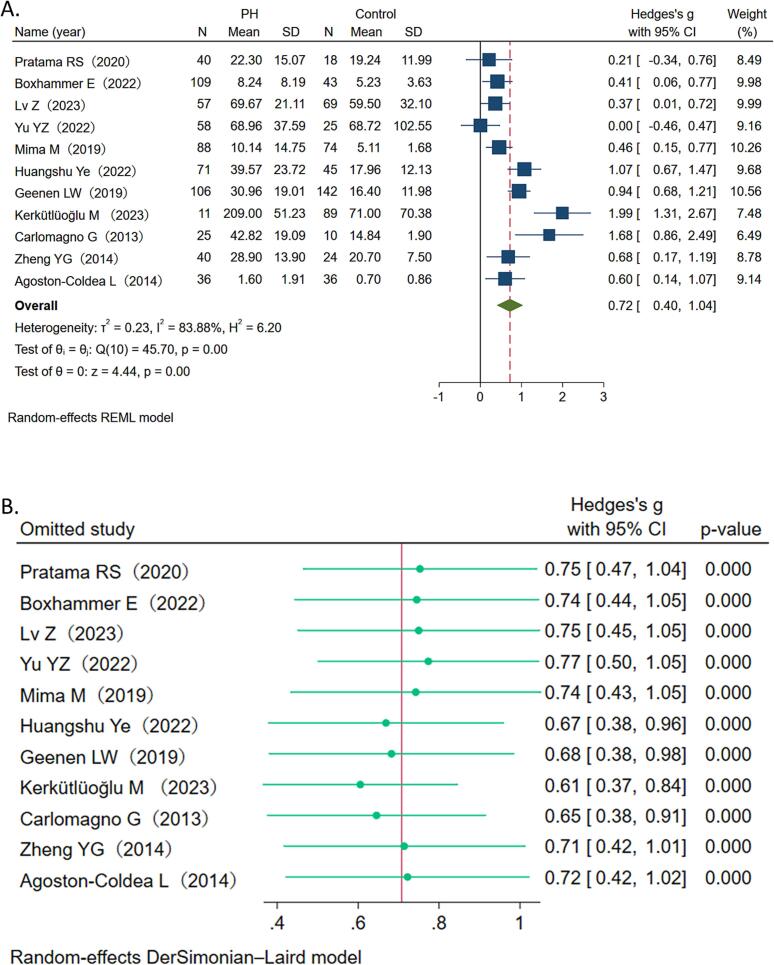
A. Differences of sST2 between control and PH groups. B. Leave-One-Out sensitivity analysis for the pooled effect size.

Eleven studies with a total of 1216 adult subjects had results of sST2 between control and PH groups [7], [8], [11], [12], [13], [14], [16], [17], [18], [21], [22]. PH of subjects composed of the researches were enrolled in the Table. The characteristics of control group were listed in the Table 2. Levels of sST2 were significantly higher in PH group (641 subjects) than control group (575 subjects) and Effect size (Hdege's g) was 0.72 (95%CI 0.40–1.04, *P* < 0.001, Fig. 3 A). To assess the robustness of our findings, a sensitivity analysis was performed using the leave-one-out method. The results confirmed the stability of our findings, with no single study significantly altering the overall effect size (Fig. 3 B).

In this meta-regression analysis, we used four variables—PH group, pH ascertainment method, sST2 assay type and clinical context—as predictors to investigate their contributions to the heterogeneity in effect sizes. The results showed that the coefficient for the clinical context variable was 0.247, which was statistically significant (*P* = 0.006, supplementary table 3), indicating that clinical context significantly contributed to the heterogeneity between studies. The sST2 assay type and PH ascertainment method variables had coefficients of 0.084 (*P* = 0.749) and − 0.098 (*P* = 0.940), respectively, neither of which were statistically significant and contributed to the heterogeneity substantially. For the PH group variable, although the coefficient was −0.018 (P = 0.940), which was not statistically significant, pH group did help reduce heterogeneity within subgroups in the subgroup analysis. This suggested that, while classification did not show a significant effect in the meta-regression, it still had some influence on heterogeneity within different study groups.

**Fig. 4:**
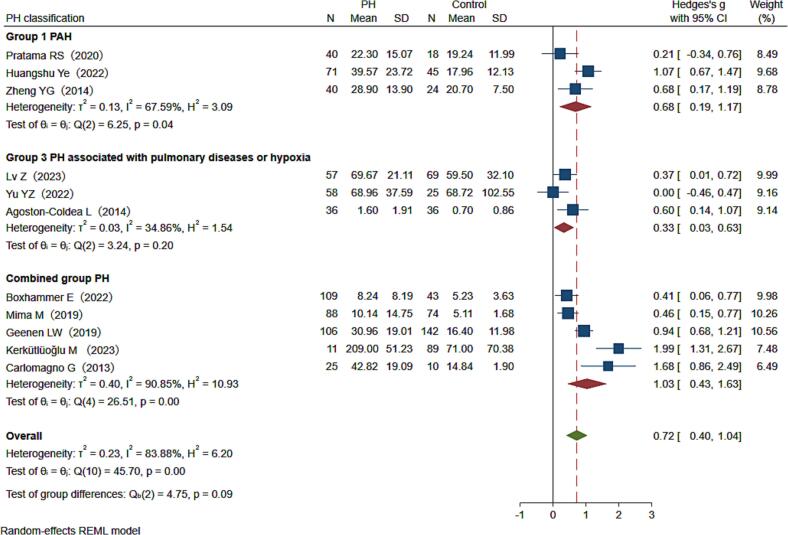
Differences of sST2 between control and PH groups using subgroup analysis.

We conducted a subgroup analysis based on the PH classifications (Fig. 4). Three subgroups were specifically defined: Group 1 (PAH), Group 3 (pH associated with pulmonary diseases or hypoxia), and Group 1–5 (combined PH classifications). Among subgroup 1 including patients with group 1 PAH, the pooled effect size for this subgroup was 0.68 (95% CI 0.19–1.17), with a heterogeneity of I^2^ = 67.6% and *P* = 0.04, indicating moderate heterogeneity. Subgroup 2 comprised three studies among PH associated with lung diseases or hypoxia. The pooled effect size for this subgroup was 0.33 (95%CI 0.03–0.63), with a decreased heterogeneity (I^2^ = 34.9%, *P* = 0.20), which suggested none of significant heterogeneity. Subgroup 3 included five studies on PH that did not focus on a specific classification. The pooled effect size for this subgroup was 1.03 (95% CI 0.43–1.63), with a high level of heterogeneity (I^2^ = 90.9%, *P* < 0.001). The heterogeneity between the groups was statistically significant, which could imply that the PH classification was a potential source of the heterogeneity.

### 3.3 Predictive value of sST2

**Fig. 5:**
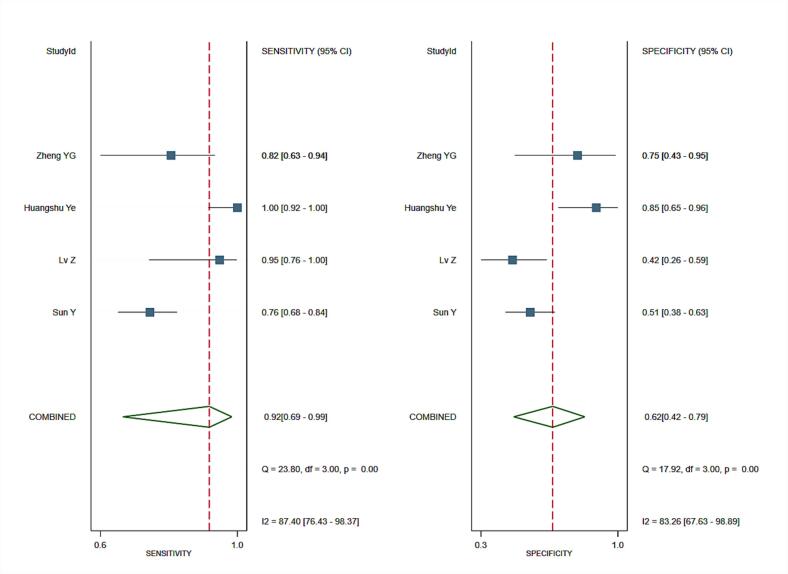
Pooled sensitivity and specificity of sST2 levels in predicting poor clinical outcomes.

**Fig. 6:**
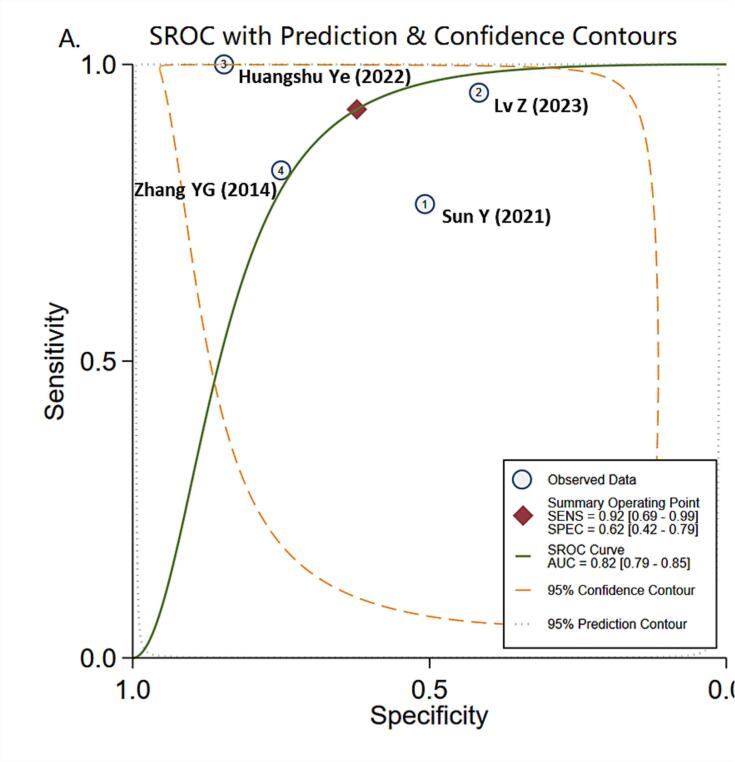
Summary of receiver operating characteristic curve plotting of accuracy of sST2.

**Fig. 7:**
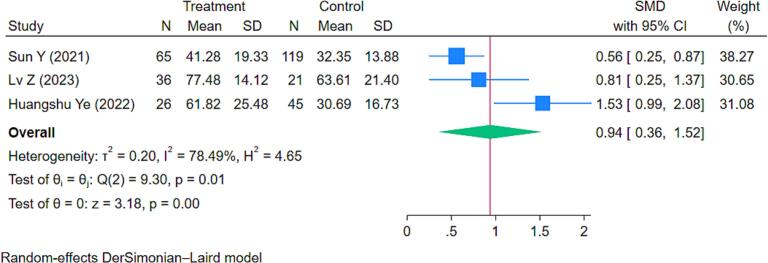
Differencf sST2 levels among patients with or without poor clinical outcomes.

Four studies included data of the sensitivity and specificity of sST2 levels in predicting clinical worsening [10], [12], [14], [21]. The cut-off value of sST2 and the definition of clinical worsening were listed in table 2. Overall, among 352 patients the sST2 had a pooled sensitivity of 0.92 (95% CI 0.69–0.99) and a pooled specificity of 0.62 (95%CI 0.42–0.79) in predicting clinical worsening (Fig. 5). The SROC curve of sST2 was plotted and area under curve (AUC) was 0.82 (95%CI 0.79–0.85) (Fig. 6). The β value and its 95% confidence interval in the HSROC model were − 0.68 (−2.11–0.76), with a Z value of −0.93 and a corresponding *P* value of 0.353, indicating that the SROC curve was symmetric. The effect measure Lambda, which reflected the diagnostic test's discriminatory ability, had an estimated value of 2.48 (95%CI: 0.42–4.54), suggesting high accuracy (supplementary table 4). Three studies reported the difference of sST2 levels between patients with and without clinical worsening [10], [12], [14]. Levels of sST2 in non-worsening group were significantly lower compared with that in the worsening group (SMD 0.94, 95%CI 0.36–1.52; *P* = 0.001, Fig. 7).

### 3.4 Correlation between sST2 and poor outcomes

**Fig. 8:**
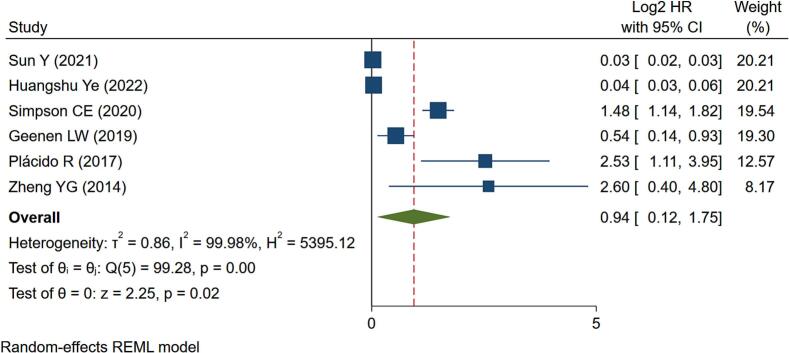
Pooled Log_2_(Hazard ratios) for sST2 and poor outcomes in pulmonary hypertension.

**Fig. 9:**
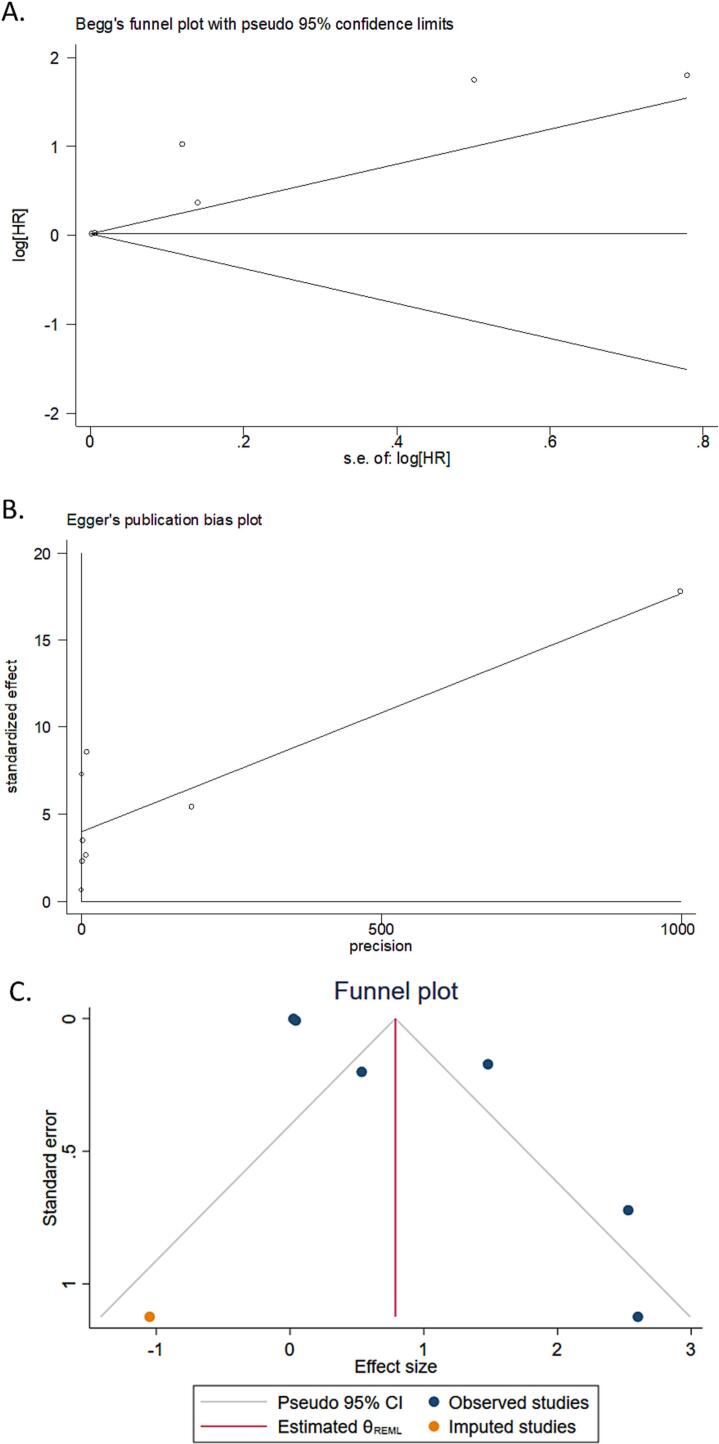
Assessment of Publication Bias.

**Fig. 10:**
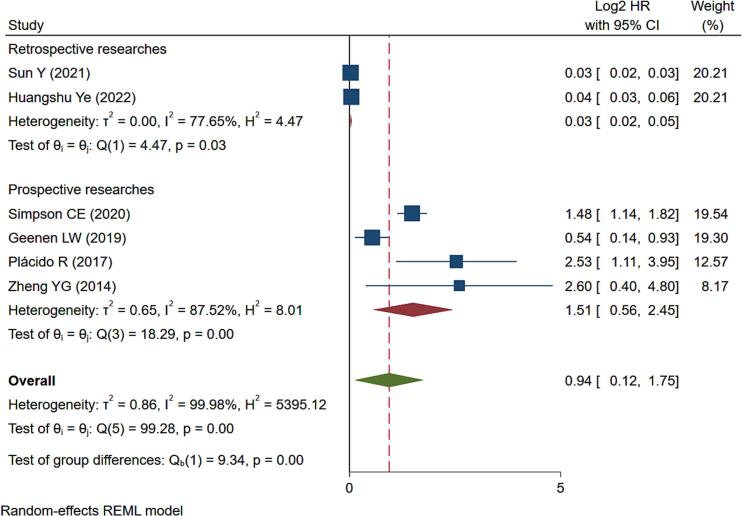
Pooled Log_2_(Hazard ratios) for sST2 and poor outcomes in pulmonary hypertension using subgroup analysis.

Our meta-analysis examined six studies that assessed the relationship between high sST2 concentrations and clinical worsening outcomes [10], [14], [15], [16], [19], [21]. The data were analyzed using Cox regression and the results were pooled up. We found that elevated sST2 levels were associated with a significant increase in the risk of poor outcomes (log_2_ HR: 0.94, 95% CI: 0.12–1.72, *P* < 0.001, Fig. 8). High heterogeneity was noted among the studies (I^2^ = 99.98%). Publication bias was evaluated according Begg and Mazumdar rank correlation (Kendall's Score = 5, *P* = 0.35, Fig. 9. A) and Egger's test (*t* = 3.32; *P* = 0.029, Fig. 9. B). A trim-and-fill analysis was conducted to analysis the publication bias as well (Fig. 9. C). To investigate the source of heterogeneity, we conducted a subgroup evaluation based on the design of the studies. Group 1 consisted of two retrospective studies, while Group 2 comprised studies that were prospective. A decrease of heterogeneity was observed among group 1 (I^2^ = 77.65%, *P* = 0.03, Fig. 10).

### 3.5 Correlation between sST2 and PAP

**Fig. 11:**
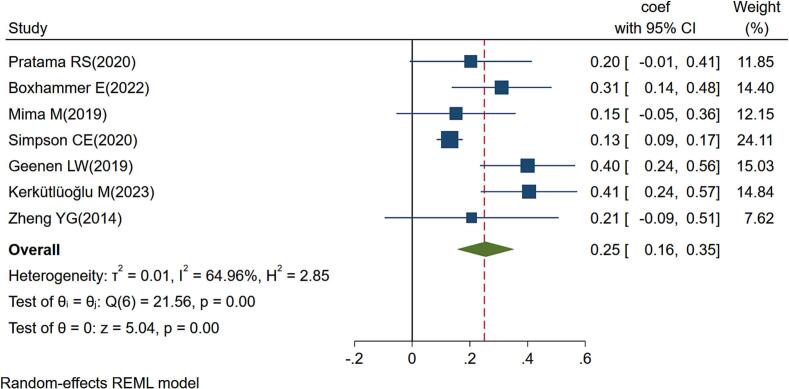
Pooled correlation coefficient of sST2 levels and PAP.

**Fig. 12:**
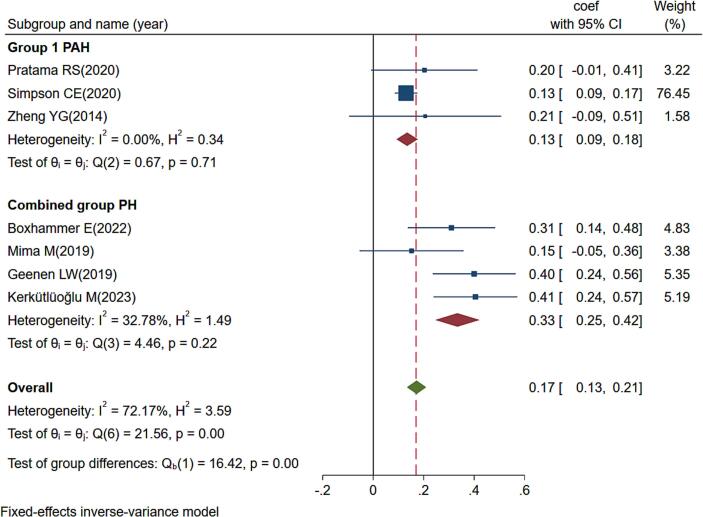
Subgroup analysis of pooled correlation coefficient of sST2 levels and PAP.

Seven studies reported the correlation between sST2 levels and mPAP using RHC [7], [8], [11], [15], [16], [17], [21]. Levels of sST2 were significantly relative to PAP and the pooled correlation coefficient was 0.25 (95%CI 0.16–0.35). Heterogeneity testing showed I^2^ = 65.0% (Fig. 11). We conducted a subgroup analysis based on patients' PH classifications: Subgroup 1 included Group 1 PAH, while Subgroup 2 consisted of combined group of PH. Our findings identified a significant correlation between sST2 levels and PAP in Group 1 pH patients. The correlation coefficient was 0.13, with a 95%CI ranging from 0.09 to 0.18, and the heterogeneity among these studies was minimal (I^2^ = 0.0%, H^2^ = 0.34, *P* = 0.71, Fig. 12). Among subgroup 2 with combined group PH, the heterogeneity was observed decrease significantly (I^2^ = 32.78%, H^2^ = 1.49, *P* = 0.22, Fig. 12). These results suggested that differences in classification contributed to the observed heterogeneity (Fig. 12).

### 3.6 Correlation between sST2 and dysfunctional right cardiac parameters

**Fig. 13:**
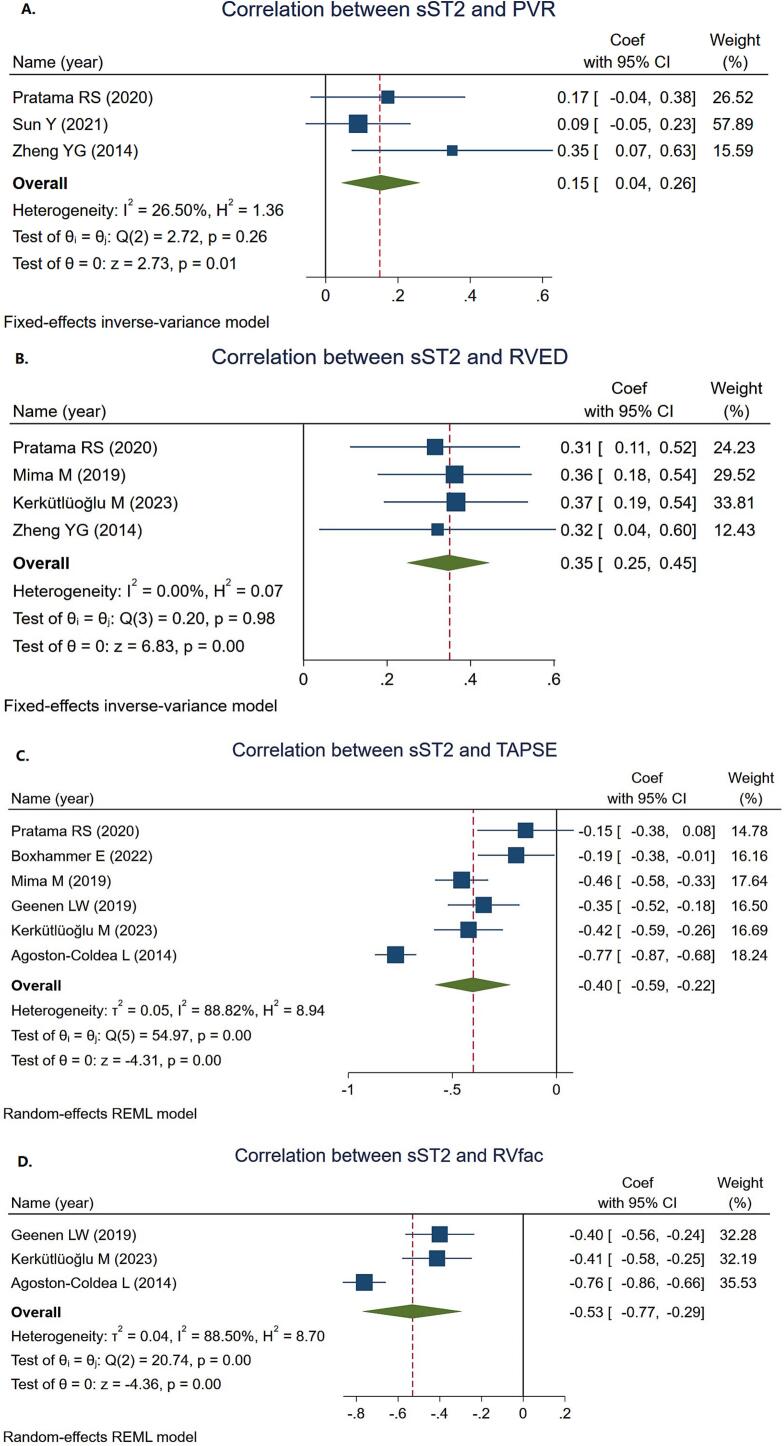
A. Correlation between sST2 and Pulmonary vascular resistance (PVR). B. Correlation between sST2 and right ventricular end-diastolic diameter (RVED). C. Correlation between sST2 and Tricuspid annular plane systolic excursion (TAPSE). D. Correlation between sST2 and right ventricular fractional area change (RVfac).

Three studies reported the correlation between sST2 levels and Pulmonary vascular resistance (PVR). We pooled the results that showed correlation coefficient was 0.15 (95%CI 0.04–0.26) with a low heterogeneity of I^2^ = 26.5% (Fig. 13 A) [8], [10], [21]. Four studies reported the correlation between sST2 and right ventricular end-diastolic diameter (RVED). The pooled effect size was 0.35 (95%CI 0.25–0.45) which indicated that sST2 was positively correlated with RVED (Fig. 13 B) [7], [8], [17], [21]. Six studies reported the correlation between sST2 and Tricuspid annular plane systolic excursion (TAPSE). The pooled results revealed effect size was −0.40 (95%CI -0.59- -0.22 Fig. 13 C) [7], [8], [11], [16], [17], [22]. Three studies reported the correlation between sST2 and right ventricular fractional area change (RVfac). The pooled effect size was −0.53 (95%CI -0.77- -0.29 Fig. 13 D) [16], [17], [22].

### 3.7 The correlation between sST2 and clinical parameters

**Fig. 14:**
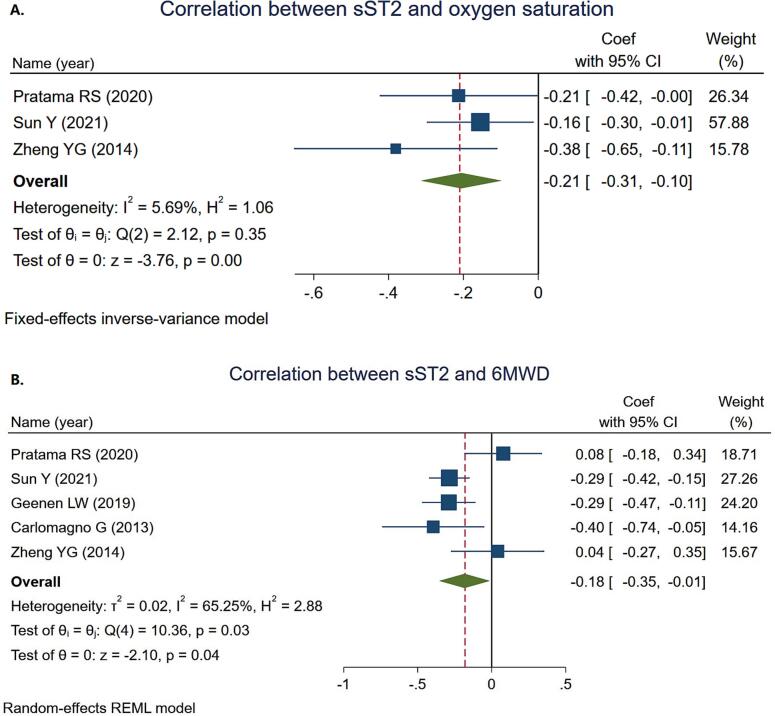
A. Correlation between sST2 and Oxygen saturation. B. Correlation between sST2 and 6-min-walk distance (6MWD).

Three studies reported the correlation between sST2 and oxygen saturation. The pooled results demonstrated that a negative correlation was found, with an effect size of −0.21 (95%CI -0.31- -0.10) [8], [10], [21] (Fig. 14A). Five studies reported the correlation between sST2 and 6-min walk distance (6MWD). The pooled results showed that sST2 was significantly correlated with the 6-min walk distance, with an effect size of −0.18 (95%CI -0.35- -0.01) [8], [10], [16], [18], [21] (Fig. 14B).

## 4 Discussion

SST2 is a biomarker increasingly recognized for its clinical practice in the diagnosis and management of various cardiovascular diseases [23]. High levels of sST2 are associated with poor outcomes in heart failure patients, including increased risk of mortality and hospitalization. Monitoring sST2 levels can help predict the risk of these events [24]. Additionally, changes in sST2 levels can reflect the response to therapy in heart failure management. As research continued, the applications of sST2 expanded to management of several other diseases including PH, renal disorders and tumor [25].

PH is a kind of progressive diseases and presents a significant clinical challenge in therapeutic and diagnostic fields due to the non-specific symptoms exhibited by patients. Additionally, pH is a life-threatening condition associated with increased mortality regardless of the classification and underlying etiology. It was reported that PH patients had survival rates of between 68% and 93% at 1 year and 39% and 77% at 3 years [26]. Therefore, timely diagnosis and therapy are crucial for improving long-term outcomes and preventing disease progression. It is emphasized to find an accessible and applicable biomarker for PH to enhance outcomes.

Several studies reported that elevated levels of sST2 in patients with PH. The association between higher sST2 levels and poor outcomes was also reported [10], [12], [14]. Despite these findings, the correlation between levels of sST2 and PAP remained inconsistent across different studies [7], [21]. Our research explored and discussed the effectiveness of sST2 as indicator for PH management. In this meta-analysis, our findings demonstrated a significant correlation between sST2 and PAP (r 0.26, 95%CI 0.14–0.38, *P* = 0.001). We also assessed the prognostic predictive value of sST2 among patients with PH. Elevated sST2 levels were consistently observed in patients with PH (SMD: 0.71, 95%CI 0.43–0.98, *P* < 0.001) and were associated with poor outcomes including readmission, heart failure, lung transplantation and death (ln HR: 0.08, 95% CI 0.03–0.14, *P* = 0.001). Four studies incorporated into our research constructed Receiver Operating Characteristic (ROC) curves to investigate the value of sST2 in predicting the prognosis of PH. We pooled up the data from these studies, revealing good sensitivity and specificity in our findings. The sensitivity and specificity were remarkably high at 0.92 (95% CI: 0.69–0.99, Q = 23.8, *P* < 0.001) and 0.62 (95%CI: 0.42–0.79, Q = 17.92, *P* < 0.001) respectively. Additionally, by plotting the Summary ROC (SROC), we obtained an area under the curve (AUC) of 0.82. This indicated that sST2 was a effective biomarker for predicting the prognosis of PH. SST2 can probably contribute to categorize patients into different risk groups based on sST2 levels and help clinicians make individualized treatment plans. It was reported that sST2 was more sensitive and superior than N-terminal pro-brain natriuretic peptide (NT-proBNP) as a biomarker for PH [16], [27]. Another advantage of sST2 is that researches have shown no correlation between sST2 levels and factors such as age, sex, BMI, or comorbidities, suggesting that sST2 levels are not significantly influenced by these variables. Therefore, changes in sST2 levels can directly reflect the underlying condition, independent of demographic factors [11], [15], [16]. Consequently, monitoring sST2 levels can help clinicians assess the effectiveness of treatment and the progression of the disease. An increase in sST2 over time indicates elevation of PAP and worsening of the disease, so that clinicians would need to adjust therapy, such as the intensification of pulmonary arterial hypertension-specific treatments or the initiation of supportive therapies like diuretics and oxygen [24], [28].

We included 15 studies in our research to validate the role of sST2 in management of PH. Compared with control group, increased sST2 levels were found in PH group (SMD 0.71, 95%CI 0.44–0.99, *P* < 0.001). Elevated sST2 levels in patients suspected of having PH can aid in the diagnosis, as they indicate cardiac stress due to increased pulmonary pressures [29]. However, the results showed a substantial heterogeneity and we conducted the meta-regression and subgroup analysis to explore the source. In this meta-regression analysis, we included four variables—PH group, pH ascertainment method, sST2 assay type and clinical context—as predictors to explore their contributions to the heterogeneity in effect sizes. The clinic context, which reflects the characteristics of the populations included in the studies, showed a significant contribution to heterogeneity (coefficient = 0.247, *P* = 0.006). The variable's strong influence on heterogeneity was primarily due to the fact that studies in the meta-analysis often included populations with distinct or combined characteristics, and there were relatively few studies with similar patient profiles. As a result, the clinic context variable had a prominent role in explaining the heterogeneity across studies. However, when performing subgroup analyses based on the clinic variable, each subgroup typically contained only one or two studies. This limitation led us to explore subgroup analyses using the group variable. The group variable represents the classification of patients included in each study. Specifically, it categorizes the studies into three groups: (1) studies focused on PAH patients, (2) studies focused on PH associated with pulmonary diseases or hypoxia, and (3) studies including various types of PH patients. Although the PH group variable did not show a significant contribution to heterogeneity in the meta-regression, it served as a simple way to summarize the population characteristics of each study. We decided to perform subgroup analysis based on the group variable because it effectively captured the different patient populations across studies. The results of the subgroup analysis using the PH group indicated a reduction in heterogeneity within the subgroups. This suggestted that, although the PH classification was not a significant source of heterogeneity in the meta-regression, it still helped to better define the populations and reduce some of the heterogeneity when studies with similar patient groups were analyzed together.

Among the patients with group 1 PAH, a mild heterogeneity was observed. sST2 levels were significant elevated among PH patients (SMD: 0.69, 95%CI 0.19–1.19, *P* = 0.006) and correlated with PAP (r: 0.13, 95%CI 0.09–0.18, *P* < 0.001). Idiopathic PAH (IPAH) emerges as the most common subtype among PAH cases, accounting for 50–60% of all cases. It is followed by PAH associated with connective tissue disease (CTD), congenital heart disease (CHD), and porto-pulmonary hypertension (PoPH) [30]. Despite the availability of various medications such as endothelin receptor antagonists, phosphodiesterase inhibitors and prostacyclin analogues for the treatment of PAH, none can achieve a complete cure. The primary goal of current treatments is to alleviate symptoms and control disease progression [31], [32]. Therefore, early diagnosis and timely recognition of disease changes are particularly crucial. Further subclassification of PAH and exploring the practical value of sST2 in these subtypes remain critical for future researches.

In subgroup2, pH secondary to lung disease or hypoxemia, levels of sST2 were significantly elevated compared to control groups (SMD: 0.33, 95%CI 0.02–0.64, *P* = 0.04) with no significant heterogeneity (I^2^ = 38.29, *P* = 0.20). SST2 can potentially serve as a prognostic predictor in this group. The main cause of this group of PH is chronic obstructive pulmonary disease (COPD). PH was related to higher hospital mortality, length of stay, readmission and costs among COPD patients [33]. Urban et al. reported that sST2 was an independent predictor of all-cause mortality in patients with COPD. The concentrations of sST2 were associated with the severity of disease and long-term outcome in patients with COPD [34]. However, this type of PH often did not respond well to medications reducing PAP, and the main treatment focused on primary disease [35], [36]. Given the correlation between sST2 levels and PAP and cardiac function, regular assessment of sST2 levels can contribute to timely adjustment of treatment to prevent complications or heart events. Additionally, the study design probably was also a source of heterogeneity when it came to the correlation between sST2 and poor outcomes. Subgroup analysis grouped between retrospective and prospective studies, demonstrated a reduction in heterogeneity, although it remained substantial (Fig. 10).

This meta-analysis demonstrated that elevated sST2 levels were significantly associated not only with increased PAP but also with impaired clinical parameters including oxygen saturation, 6-min walk distance (6MWD), right ventricular function (TAPSE, RVfac), right ventricular structural remodeling (RVED), and pulmonary vascular resistance (PVR) in patients with PH. These findings suggested that sST2 played a crucial role in the pathophysiology of PH, potentially served as a multidimensional biomarker reflecting integrated cardiopulmonary pathophysiology, encompassing right ventricular dysfunction, right ventricular overload, and functional exercise capacity limitations. That was consistent with recent study results [37], [38]. In chronic heart failure, a meta-analysis of outpatient cohorts revealed that elevated sST2 independently predicts all-cause and cardiovascular mortality (HR 1.75 per doubling for all-cause mortality), with its predictive accuracy being highest when guideline-directed therapy is optimized [39]. In acute heart failure, a pooled analysis found that both admission and discharge sST2 levels predict all-cause and cardiovascular mortality, with discharge levels also serving as indicators for heart failure readmission [40]. Furthermore, an individual patient-data meta-analysis showed that sST2 provides additional prognostic information beyond NT-proBNP and high-sensitivity troponin T, supporting the use of a multi-marker approach [41]. Our correlation of sST2 with hemodynamic parameters like RVED and PVR further underscores the role of sST2 in right heart failure and its potential to guide therapeutic interventions aimed at reducing right ventricular strain in PH. The correlations between sST2 and blood oxygen saturation and 6MWD reflect its association with disease severity and prognosis, including factors like hypoxia and exercise tolerance [42]. Given the potential for sST2 to reflect not only pulmonary vascular resistance and the underlying pathophysiology of right ventricular dysfunction but also the prognosis of PH, it can serve as a valuable biomarker for monitoring disease progression and guiding treatment decisions in PH. However, recent studies have indicated that changes in sST2 levels may not be predictive of survival or right heart failure [37]. Therefore, further research is essential to explore the optimal use of sST2 in clinical practice, particularly in understanding its role in managing right heart failure and PH.

Although Begg's test showed no significant bias, the results from the Trim-and-Fill method suggest that publication bias did influence the effect size estimate (Fig. 9). The initial effect size was 0.937 (95%CI 0.120–1.753), indicating a positive and statistically significant effect. However, after applying the Trim-and-Fill method to account for the potential underrepresentation of studies with small or null effects, the effect size decreased to 0.788 (95%CI 0.003–1.579). This adjustment highlighted the overestimation of the effect size due to publication bias, as smaller studies with null results were likely omitted, leading to an inflated original estimate.

This study still had several limitations as well. Firstly, there was considerable inevitable heterogeneity, which was probably attributed to variations in PH classification and the methods used to measure sST2. Moreover, the studies included in our analysis consisted of retrospective designs, and there were variations in observational endpoints, though these studies did not show significant publication bias. Additionally, this study observed a potential gender bias in the incidence of PH, particularly in PAH and CTD-PH, where females were more commonly affected. However, the lack of gender-specific data in the studies included made it challenging to assess the impact of gender on sST2's clinical utility in PH management, though researchers suggested gender did not significantly affect the effect size [11], [16].

## 5 Future direction

Further research is needed to clarify the precise role of sST2 in managing PH. Further studies should focus on specific PH classification systems to minimize the heterogeneity observed in previous analyses, as well as standardizing the methods used to measure sST2 levels. Large-scale, prospective, multi-center trials are crucial to validate the prognostic value of sST2 in various PH groups, particularly in those with pulmonary arterial hypertension (PAH) and connective tissue disease-related PH (CTD-PH). Additionally, integrating sST2 levels with advanced imaging techniques and functional parameters could significantly improve the precision of risk stratification frameworks, enabling more accurate identification of high-risk patients. Exploring the utility of sST2 in monitoring treatment response and guiding therapeutic strategies, especially when used in conjunction with other biomarkers and clinical factors, could enhance personalized treatment approaches for PH. Ultimately, the goal is to establish sST2 as a reliable, actionable biomarker that informs clinical decision-making and optimizes patient outcomes.

## 6 Conclusion

SST2 levels were elevated among patients with PH and correlated to PAP and right cardiac dysfunctional parameters. SST2 played a reliable role in predicting prognosis of PH despite the limitations and heterogeneity among the studies. Therefore, sST2 can assist clinicians identify the patients with PH who are at high risk of poor outcomes.

## CRediT authorship contribution statement

**Liuzhao Cao:** Writing – review & editing, Writing – original draft, Supervision, Software, Methodology, Investigation, Formal analysis, Data curation, Conceptualization. **Weiyun Teng:** Writing – review & editing, Writing – original draft, Visualization, Validation, Supervision, Software, Methodology, Formal analysis, Data curation, Conceptualization. **Linli Sang:** Writing – review & editing, Supervision, Methodology, Investigation, Formal analysis, Data curation. **Xingxiang Xu:** Writing – review & editing, Visualization, Validation, Supervision, Methodology, Formal analysis, Data curation, Conceptualization.

## Ethical statement

This systematic review and meta-analysis was conducted in accordance with ethical guidelines. As the study involves the analysis of previously published data and does not involve primary data collection, no ethics committee approval was required. All studies included in this review were approved by the relevant ethical committees of their respective institutions, and informed consent was obtained from participants in the original studies.

## Declaration of competing interest

The authors declare that they have no known competing financial interests or personal relationships that could have appeared to influence the work reported in this paper.
