# Supplementary material for: The role of soluble suppression of tumorigenicity 2 in pulmonary hypertension: a systematic review and meta-analysis
Source: Am Heart J Plus. 2026 Jul 2;68:100827. doi: 10.1016/j.ahjo.2026.100827 (PMC13377141; doi:10.1016/j.ahjo.2026.100827)
Supplement: Supplementary file 1 — Supplementary material [file mmc1.pdf]

Supplementary table 1. GRADE Assessment of the Quality of Evidence

**Name (year)**	**Risk of Bias**	**Inconsistency**	**Indirectness**	**Imprecision**	**Publication Bias**	**GRADE Rating**
Pratama RS (2020)	Moderate	Low	Low	Moderate	Low	Low
Yang HS (2016)	Low	Low	Low	Low	Low	High
Sun Y (2021)	Moderate	Low	Moderate	Low	Low	Moderate
Boxhammer E (2022)	Moderate	Low	Low	Low	Low	Moderate
Lv Z (2023)	Moderate	Low	Moderate	Moderate	Low	Low
Yu YZ (2022)	Moderate	Low	Moderate	Moderate	Low	Low
Mirna M (2019)	Moderate	Low	Low	Low	Low	Moderate
Huangshu Ye (2022)	Moderate	Low	Low	Moderate	Low	Moderate
Simpson CE (2020)	Low	Low	Low	Low	Low	High
Geenen LW (2019)	Moderate	Moderate	Low	Low	Low	Moderate
Kerkütlüoğlu M (2023)	Low	Moderate	Low	Moderate	Low	Moderate
Carlomagno G (2013)	High	Moderate	Low	Moderate	Low	Moderate
Plácido R (2017)	Moderate	Low	Low	Moderate	Low	Moderate
Zheng YG (2014)	Moderate	Low	Low	Moderate	Low	Moderate
Agoston-Coldea L (2014)	Moderate	Moderate	Low	Moderate	Low	Moderate

Supplemental Table 2: Ratings for included studies using the Quality In Prognosis Studies (***QUIPS***) *tool*

**Name (year)**	**Study participation**	**Study attrition**	**Prognostic factor measurement**	**Outcome measurement**	**Study Confounding**	**Statistical Analysis and reporting**	**Risk of Bias Total**
Lv z (2023)	Moderate	Moderate	Low	Moderate	Moderate	Low	Moderate
Ye HS(2022)	Moderate	Low	Moderate	Moderate	Low	Low	Moderate
Simpson CE (2020)	Low	Low	Low	Low	Low	Low	Low
Geenen LW (2019)	Moderate	Low	Low	Low	Low	Low	Low
Sun Y (2021)	Moderate	Low	Low	Low	Moderate	Low	Moderate
Plácido R (2016)	Moderate	Moderate	Low	Low	Moderate	Low	Moderate
Zheng YG (2014)	Moderate	Moderate	Low	Low	Moderate	Low	Moderate

Supplementary table 3. Meta-regression Analysis Results for the four virables

Meta-regression	Residual heterogeneity:
Effect-size label:Hedges's g	tau2 = .144
	I2 (%) = 76.29
	H2 = 4.22
	R-squared (%) = 37.56
	Wald chi2(4) = 9.21
	Prob > chi2 = 0.0560

	Coef.	Std. Err.	z	P>z	[95% Conf.Interval]	
clinical context	0.248	0.089	2.77	0.006	0.072,	0.422
sST2 assay type	0.083	0.261	0.32	0.749	-0.428,	0.596
PH assertainment method	-0.098	0.3	-0.33	0.744	-0.685,	0.490
PH group	-0.018	0.247	-0.08	0.94	-0.500,	0.463
_cons	-0.274	0.653	-0.42	0.674	-1.555,	1.006

Supplementary table 4. Meta-analysis of predictive accuracy

	Coef.	Std. Err.	z	P>z	[95% Conf.Interval]	
Bivariate						
E(logitSe)	2.50	0.86			0.81	4.19
E(logitSp)	0.50	0.41			-0.31	1.31
Var(logitSe)	1.85	2.34			0.15	22.11
Var(logitSp)	0.47	0.49			0.06	3.62
Corr(logits)	0.74	0.47			-0.79	0.99
HSROC						
Lambda	2.48	1.05			0.42	4.54
Theta	0.54	0.39			-0.22	1.30
beta	-0.68	0.73	-0.93	0.35	-2.11	0.76
s2alpha	3.25	3.20			0.47	22.31
s2theta	0.12	0.23			0.00	4.50
Summary pt.						
Se	0.92	0.06			0.69	0.99
Sp	0.62	0.10			0.42	0.79
DOR	20.11	22.55			2.23	181.10
LR+	2.45	0.72			1.38	4.35
LR-	0.12	0.11			0.02	0.69
1/LR-	8.22	7.26			1.46	46.41

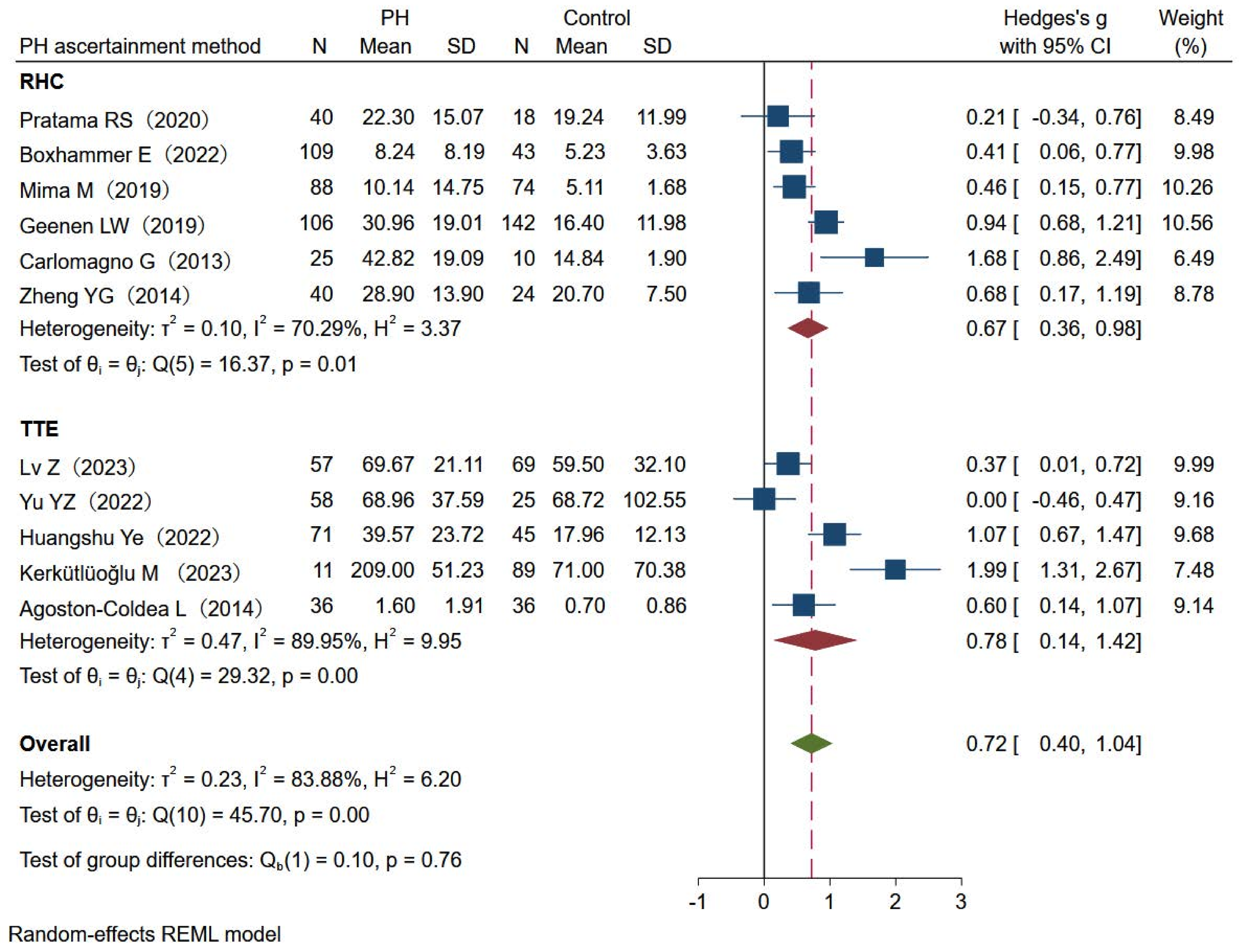
PH ascertainment method | PH N | PH Mean | PH SD | Control N | Control Mean | Control SD | Hedges's g with 95% CI | Weight (%)
RHC
Pratama RS (2020) 40 22.30 15.07 18 19.24 11.99 0.21 [-0.34, 0.76] 8.49
Boxhammer E (2022) 109 8.24 8.19 43 5.23 3.63 0.41 [0.06, 0.77] 9.98
Mima M (2019) 88 10.14 14.75 74 5.11 1.68 0.46 [0.15, 0.77] 10.26
Geenen LW (2019) 106 30.96 19.01 142 16.40 11.98 0.94 [0.68, 1.21] 10.56
Carlomagno G (2013) 25 42.82 19.09 10 14.84 1.90 1.68 [0.86, 2.49] 6.49
Zheng YG (2014) 40 28.90 13.90 24 20.70 7.50 0.68 [0.17, 1.19] 8.78
Heterogeneity: τ^2 = 0.10, I^2 = 70.29%, H^2 = 3.37 0.67 [0.36, 0.98]
Test of $\theta_i = \theta_j$: Q(5) = 16.37, p = 0.01
TTE
Lv Z (2023) 57 69.67 21.11 69 59.50 32.10 0.37 [0.01, 0.72] 9.99
Yu YZ (2022) 58 68.96 37.59 25 68.72 102.55 0.00 [-0.46, 0.47] 9.16
Huangshu Ye (2022) 71 39.57 23.72 45 17.96 12.13 1.07 [0.67, 1.47] 9.68
Kerkütlüoğlu M (2023) 11 209.00 51.23 89 71.00 70.38 1.99 [1.31, 2.67] 7.48
Agoston-Coldea L (2014) 36 1.60 1.91 36 0.70 0.86 0.60 [0.14, 1.07] 9.14
Heterogeneity: τ^2 = 0.47, I^2 = 89.95%, H^2 = 9.95 0.78 [0.14, 1.42]
Test of $\theta_i = \theta_j$: Q(4) = 29.32, p = 0.00
Overall 0.72 [0.40, 1.04]
Heterogeneity: τ^2 = 0.23, I^2 = 83.88%, H^2 = 6.20
Test of $\theta_i = \theta_j$: Q(10) = 45.70, p = 0.00
Test of group differences: $Q_b(1)$ = 0.10, p = 0.76
-1 0 1 2 3
Random-effects REML model

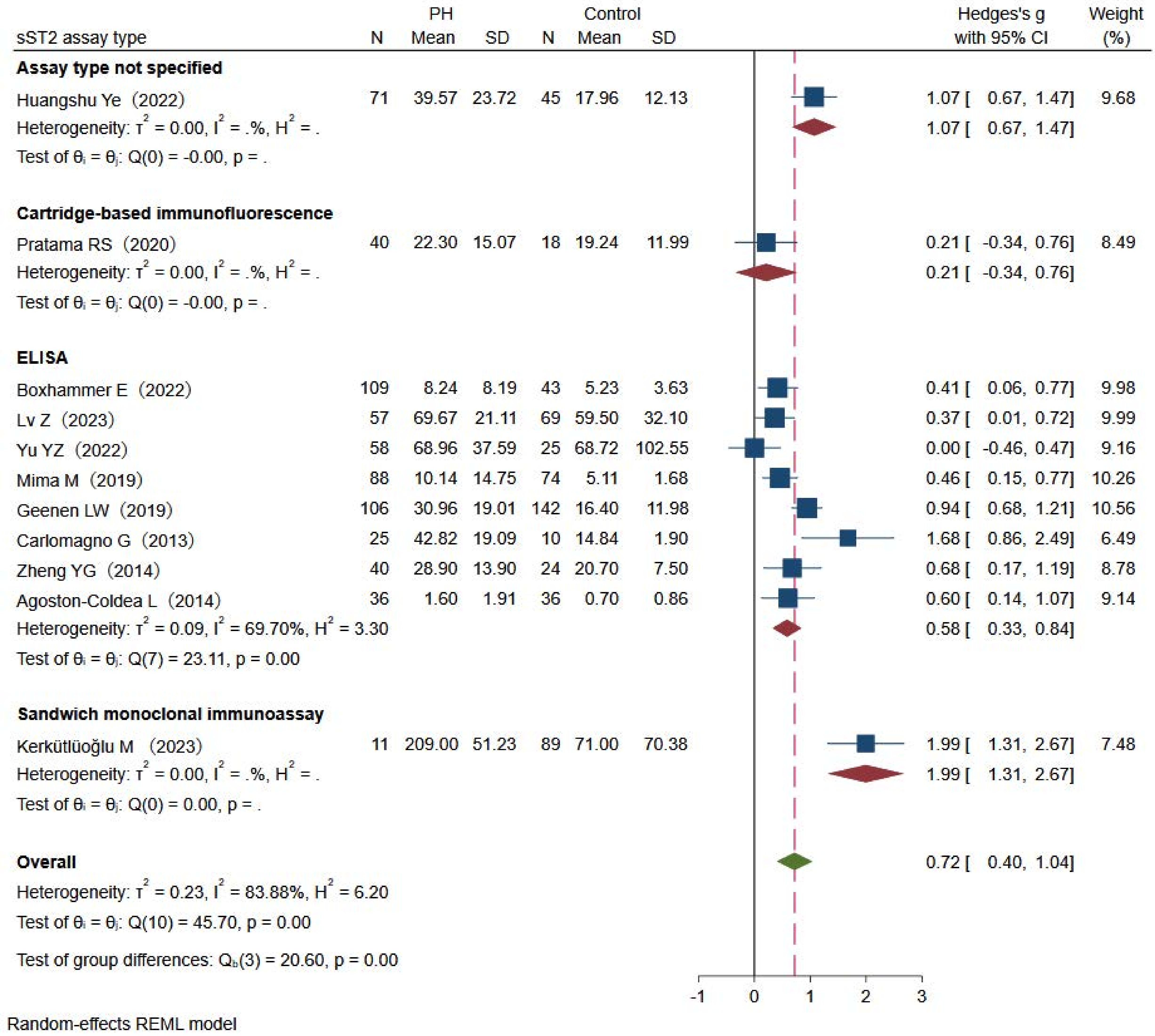

PH
Control
sST2 assay type N Mean SD N Mean SD
Hedges's g with 95% CI
Weight (%)
Assay type not specified
Huangshu Ye (2022) 71 39.57 23.72 45 17.96 12.13 1.07 [0.67, 1.47] 9.68
Heterogeneity: τ^2 = 0.00, I^2 = .%, H^2 = . 1.07 [0.67, 1.47]
Test of $\theta_i = \theta_j$: Q(0) = -0.00, p = .
Cartridge-based immunofluorescence
Pratama RS (2020) 40 22.30 15.07 18 19.24 11.99 0.21 [-0.34, 0.76] 8.49
Heterogeneity: τ^2 = 0.00, I^2 = .%, H^2 = . 0.21 [-0.34, 0.76]
Test of $\theta_i = \theta_j$: Q(0) = -0.00, p = .
ELISA
Boxhammer E (2022) 109 8.24 8.19 43 5.23 3.63 0.41 [0.06, 0.77] 9.98
Lv Z (2023) 57 69.67 21.11 69 59.50 32.10 0.37 [0.01, 0.72] 9.99
Yu YZ (2022) 58 68.96 37.59 25 68.72 102.55 0.00 [-0.46, 0.47] 9.16
Mima M (2019) 88 10.14 14.75 74 5.11 1.68 0.46 [0.15, 0.77] 10.26
Geenen LW (2019) 106 30.96 19.01 142 16.40 11.98 0.94 [0.68, 1.21] 10.56
Carlomagno G (2013) 25 42.82 19.09 10 14.84 1.90 1.68 [0.86, 2.49] 6.49
Zheng YG (2014) 40 28.90 13.90 24 20.70 7.50 0.68 [0.17, 1.19] 8.78
Agoston-Coldea L (2014) 36 1.60 1.91 36 0.70 0.86 0.60 [0.14, 1.07] 9.14
Heterogeneity: τ^2 = 0.09, I^2 = 69.70%, H^2 = 3.30 0.58 [0.33, 0.84]
Test of $\theta_i = \theta_j$: Q(7) = 23.11, p = 0.00
Sandwich monoclonal immunoassay
Kerkütlüoğlu M (2023) 11 209.00 51.23 89 71.00 70.38 1.99 [1.31, 2.67] 7.48
Heterogeneity: τ^2 = 0.00, I^2 = .%, H^2 = . 1.99 [1.31, 2.67]
Test of $\theta_i = \theta_j$: Q(0) = 0.00, p = .
Overall 0.72 [0.40, 1.04]
Heterogeneity: τ^2 = 0.23, I^2 = 83.88%, H^2 = 6.20
Test of $\theta_i = \theta_j$: Q(10) = 45.70, p = 0.00
Test of group differences: $Q_b(3)$ = 20.60, p = 0.00
-1
0
1
2
3
Random-effects REML model
